# Candidate metastasis suppressor genes uncovered by array comparative genomic hybridization in a mouse allograft model of prostate cancer

**DOI:** 10.1186/1755-8166-2-18

**Published:** 2009-09-26

**Authors:** Yajun Yi, Srinivas Nandana, Thomas Case, Colleen Nelson, Tatjana Radmilovic, Robert J Matusik, Karen D Tsuchiya

**Affiliations:** 1 Department of Medicine, Vanderbilt University Medical Center, Nashville, TN, USA; 2 Department of Urologic Surgery and Vanderbilt Ingram Cancer Center, Vanderbilt University Medical Center, Nashville, TN, USA; 3 Department of Urologic Sciences, University of British Columbia, The Prostate Centre at Vancouver General Hospital, Vancouver, British Columbia, Canada; 4 Clinical Research Division, Fred Hutchinson Cancer Research Center and Department of Laboratories, Seattle Children's Hospital, WA, USA; 5 Department of Laboratory Medicine, University of Washington School of Medicine, Seattle, WA, USA

## Abstract

**Background:**

The purpose of this study was to identify candidate metastasis suppressor genes from a mouse allograft model of prostate cancer (NE-10). This allograft model originally developed metastases by twelve weeks after implantation in male athymic nude mice, but lost the ability to metastasize after a number of *in vivo *passages. We performed high resolution array comparative genomic hybridization on the metastasizing and non-metastasizing allografts to identify chromosome imbalances that differed between the two groups of tumors.

**Results:**

This analysis uncovered a deletion on chromosome 2 that differed between the metastasizing and non-metastasizing tumors. Bioinformatics filters were employed to mine this region of the genome for candidate metastasis suppressor genes. Of the 146 known genes that reside within the region of interest on mouse chromosome 2, four candidate metastasis suppressor genes (*Slc27a2, Mall, Snrpb*, and *Rassf2*) were identified. Quantitative expression analysis confirmed decreased expression of these genes in the metastasizing compared to non-metastasizing tumors.

**Conclusion:**

This study presents combined genomics and bioinformatics approaches for identifying potential metastasis suppressor genes. The genes identified here are candidates for further studies to determine their functional role in inhibiting metastases in the NE-10 allograft model and human prostate cancer.

## Background

Prostate cancer (PCa) is a heterogeneous disease and the ability to predict its clinical outcome is limited. Numerous chromosomal abnormalities and alterations in gene expression have been reported in PCa, yet identification of many of the specific genes that drive the progression of these tumors is still lacking. The finding of the *TMPRSS2/ETS *fusion and the overexpression of ETS transcription family members in the majority of PCa illustrates the success of utilizing a bioinformatics approach to gene discovery [1], but the consequences of many other recurrent acquired genomic alterations remain to be elucidated. Studies of human PCa are hindered by the biologic and genetic heterogeneity of this disease not only between individuals, but also within a given individual. Genetically engineered mouse models of PCa provide an *in vivo *experimental system in which tumors with the same underlying etiology can be sampled during the course of progression at defined time points. The LPB-Tag mouse model of prostate cancer is one such model that has been well characterized [2-4].

We have previously described the establishment of an allograft (NE-10) from a primary prostate tumor from the LPB-Tag mouse model that consistently metastasized by 12 weeks after transplantation in nude mice in early passages [4]. Conventional cytogenetic analysis of the NE-10 allograft revealed numeric and structural chromosome abnormalities, including a deletion of distal chromosome 2 that was consistently present over multiple *in vivo *passages of the allograft in nude mice [4]. A similar chromosome 2 deletion has also been described in a mouse model of acute promyelocytic leukemia [5]. After repeated *in vivo *passages, the NE-10 allograft eventually lost the ability to metastasize. In this study, we have taken advantage of the differential metastasizing behavior of the NE-10 allograft, arising from the same original tumor, to screen the genome for candidate genes that play a role in metastasis. High resolution genomic technology, combined with novel bioinformatics approaches, enabled us to identify different regions of chromosome imbalances between the two allograft lines, and to propose candidate metastasis suppressor genes within a region of chromosome 2 that was found to harbor a larger deletion in metastatic compared to non-metastatic tumors.

## Methods

### NE-10 allograft model

The 12T-10 line of the LPB-Tag mouse model of prostate cancer was generated using a transgene that consists of the rat probasin promoter driving the SV40 large T antigen with deletion of the small T antigen [3]. These mice develop low-grade and high-grade prostatic intraepithelial neoplasia at 2-5 month of age, with progression to invasive and metastatic, high-grade, androgen-independent carcinoma demonstrating neuroendocrine differentiation at 6-14 months of age. A primary prostate tumor from the ventral prostate of a 12T-10 transgenic mouse was used to establish an allograft model by implantation subcutaneously in male athymic nude mice [4]. After 18 weeks, the allograft was passaged to another male nude mouse and the process was repeated to establish the NE-10 line [4]. Initial passages in all mice developed grossly visible metastases to liver and micrometastases to lung by twelve weeks after implantation. All metastases from the allografts were histologically similar to the metastasis seen in the 12T-10 mice. Later allograft passages showed histologically identical features to the early passages; however, fewer allografts developed metastases. By passage 15, metastatic potential was completely lost, at least up to the point where it was no longer feasible to maintain the mice due to the size of the subcutaneous tumors. Tumors consisting of non-metastasizing subcutaneous allografts (SQnon-met), metastasizing subcutaneous allografts (SQmet), and liver metastases (LiverMet) were collected at 12 weeks post-implantation and snap-frozen for array CGH. All procedures involving mice were approved by the Vanderbilt University Medical Center and Fred Hutchinson Cancer Research Center Institutional Animal Care and Use Committees.

### Array comparative genomic hybridization (CGH)

Array CGH was initially carried out using mouse bacterial artificial chromosome (BAC) arrays produced by the Genomics Shared Resource, Fred Hutchinson Cancer Research Center as described [6]. The mouse BAC clone set was obtained from A. Bradley, Wellcome Trust Sanger Institute [7] and provides an average resolution of 1 Mb. DNA was isolated using the Gentra Puregene genomic DNA purification kit (Qiagen, Valencia, CA). For the BAC arrays, three SQnon-met tumors (passage 19), three SQmet (passages 4, 9, and 12), and four LiverMet (two each from passages 9 and 12) were analyzed. One matched SQmet and LiverMet from the same nude mouse at passage 9 was included in the analysis. Pooled DNA obtained from normal kidney from four different CD-1 males was used as a reference. DNA labeling, hybridization, scanning, and data analysis was performed as described previously [6].

Array CGH was repeated on a subset of two tumors each from the SQnon-met and LiverMet groups using the Agilent mouse 105K oligonucleotide CGH arrays. These arrays were designed using UCSC mm7 (NCBI build 35, August, 2005), and have an average probe spacing of 15 Kb. One ug of RsaI/AluI digested DNA was labeled with either Cy3 or Cy5 using the BioPrime Array CGH genomic labeling system (Invitrogen Corp., Carlsbad, CA). Approximately 4 ug each of Cy5 labeled test (tumor) DNA and Cy3 labeled reference (normal female C57Bl/6 liver) DNA was combined with 25 ug of mouse Cot-1 (Invitrogen Corp.), Agilent 10× blocking agent and 2× hybridization buffer (Agilent Technologies, Santa Clara, CA) to a final volume of 260 ul. Hybridization and washes were carried out according to the Agilent oligo array-based CGH protocol v. 4.0. Scanning was performed on an Agilent scanner and data extraction was carried out using Agilent feature extraction software v.9.1, employing linear and Lowess normalization. Results were analyzed and chromosome plots generated using CGHanalytics software v. 6.0. The Z-score algorithm with the threshold set at 2.0 and a 2 Mb window was used for determining gains and losses. The LPB-Tag tumors were generated on an outbred CD-1 background, and we did not have non-neoplastic DNA available from the mouse whose tumor was used to establish the allograft. As C57Bl/6 reference DNA was used for the oligonucleotide array CGH experiments, gains and losses smaller than 2 Mb were ignored in order to avoid mistaking benign copy number variants between mouse strains from acquired copy number alterations in the tumors.

To compare array CGH profiles between the different groups of tumors, the Differential Gene locus MAPping software (DIGMAP version 2.0) was used to analyze array CGH data sets [8]. Using the default parameters specified in the program, DIGMAP displayed array CGH data as a heat map based on log_2 _ratio between test and reference DNA for each tumor. The chromosome regions with significantly different log_2 _ratios were marked as differential flagged regions (DFRs) by direct visualization and computational screening using a T-test based sliding window (TTSW) analysis. For this analysis, a window size of 40 genes was used and DFRs represent regions greater than 3 standard deviations from the whole genome average T score.

### Expression arrays

Expression arrays were performed on two SQnon-met tumors (passages 11b, 18c) and four SQmet tumors (passages 2, 5, 14a, 14c). Allograft passages were labeled a, b, c, etc. when the same graft passage was implanted into more than one mouse. As a control, mouse reference RNA from two postnatal day 1 mice was pooled. RNA was isolated using the RNeasy kit (Qiagen Valencia CA), including treatment with DNase. RNA was quantified using a NanoDrop (ThermoScientific). All samples analyzed had a A260/230 ratio greater than 1.8. RNA quality was analyzed on an Agilent 2100 BioAnalyzer. All samples analyzed had a 28S peak greater than 18S peak, or an RIN number greater than 7. The 16 k mouse arrays used for the study were printed at the Vancouver Microarray Facility using Operon's 16 K 70 mer oligomers printed on aminosaline slides. Ten ug of total RNA was labeled using Genisphere's Dendrimer 350 expression array detection kit for microarrays according to the manufacturer's protocol. Samples were co-hybridized with ten ug of the above described mouse reference RNA. Arrays were pre-hybridized in 5 × SSC, 0.1% SDS and 0.2% BSA for 45 minutes. Pre-hybridization buffer was washed off with 3 × 30 second water washes and a 2 minute wash in isopropanol. Slides were spun dry at 2000 rpm for 4 minutes. The samples were applied to arrays containing 60 × 22 mm lifter slips (Erie Scientific). Slides were subsequently treated as described in the Genisphere Array 350 expression array detection kit for microarrays. Arrays were scanned on a Axon 4200AL scanner (Molecular Devices). Image intensities were extracted using ImaGene V8.0 software (BioDiscovery).

To identify chromosome regions that demonstrate differential gene expression between metastatic and non-metastatic tumors, expression array datasets were analyzed between SQnon-met and SQmet mice using DIGMAP as described above for array CGH data. A TTSW genome scan was also performed as described above.

### Identification of candidate metastasis suppressor genes in chromosome 2 DFR

A total of 146 known genes in the chromosome 2 differential region of deletion (DFR) between metastatic and non-metastatic tumors (nucleotides 122,316,740-139,585,560) were retrieved from the UCSC mm7 database . Two bioinformatics filters were designed to determine which of these 146 genes might function as candidate metastasis suppressor genes. A functional filter utilized the Gene Ontology (GO) database , PubMed , and Ingenuity Pathway Analysis (IPA, ) to evaluate the 146 genes for potential metastasis suppressor function. A parallel filter employing our recently developed human cancer expression signature database (EXALT) was used to independently validate the genes as candidate metastasis suppressors *in silico *[9]. A weighted score was assigned to each gene for both filters (see Additional file 1).

### Quantitative reverse transcription (RT)-PCR

Total RNA was isolated using the RNeasy Mini kit (Qiagen, Valencia, CA) according to the manufacturer's protocol, including the recommended DNase treatment step. Three ug of RNA was reverse transcribed using the ReactionReady First Strand cDNA Synthesis Kit (SuperArray Bioscience Corp., Frederick, MD). Primer sets for mouse *Hprt1*, *Slc27a2*, *Mal*, *Snrpb*, and *Rassf2 *were purchased from SuperArray (proprietary primers, sequence not disclosed).

PCR reactions were carried out in triplicate in a 25 μL volume using SYBR Green Master Mix (SuperArray). The standard two-step amplification with an annealing temperature of 60°C was performed in an ABI 7900 PCR machine. *Hprt1 *was chosen as an endogenous control gene because *Hprt1 *expression values were stable among test samples in the microarray expression data set.

To allow for a comparison between samples and the two groups, quantities of all target genes in the test samples and a common reference RNA (Mouse XpressRef Universal Total RNA, SuperArray) were normalized to the corresponding *Hprt1 *levels. Relative expression levels (fold changes) were calculated using the relative standard curve method as outlined in the manufacturer's technical user manual (SuperArray). A standard curve was generated using the fluorescent data from 10-fold serial dilutions of the common reference RNA sample. Four tumors each from the metastasizing and non-metastasizing allografts were analyzed. Statistically significant differences in expression between the two groups were determined using a Student's T-Test.

## Results

### Differences in copy number alterations between metastatic and non-metastatic tumors

**Figure 1:**
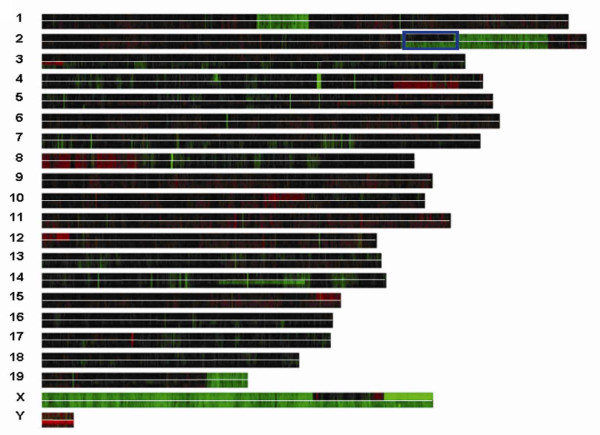
**Heat map of oligonucleotide array CGH results**. Array CGH results are partitioned by chromosome number and clustered by probe location in the chromosomes. The length of each chromosome bar is based on the total number of probes for that chromosome present on the array. Within each chromosome bar, each row represents a separate NE-10 tumor, and the non-metastatic tumors (SQnon-Met) are separated from the metastatic tumors (LiverMet) by the white line (SQnon-Met above and LiverMet below the line). Black represents normal copy number in tumor compared to the normal reference; red represents increased copy number; green represents decreased copy number. A larger interstitial deletion in chromosome 2 can be visualized in the metastatic compared to the non-metastatic tumors (blue box). Relative loss of the X chromosome in the metastatic tumors, and most of the X chromosome in the non-metastatic tumors, is seen because array CGH for these tumor samples was performed using a sex-mismatched (female) reference DNA. The non-metastatic tumors also show a region of copy number gain on the X chromosome.

**Figure 2:**
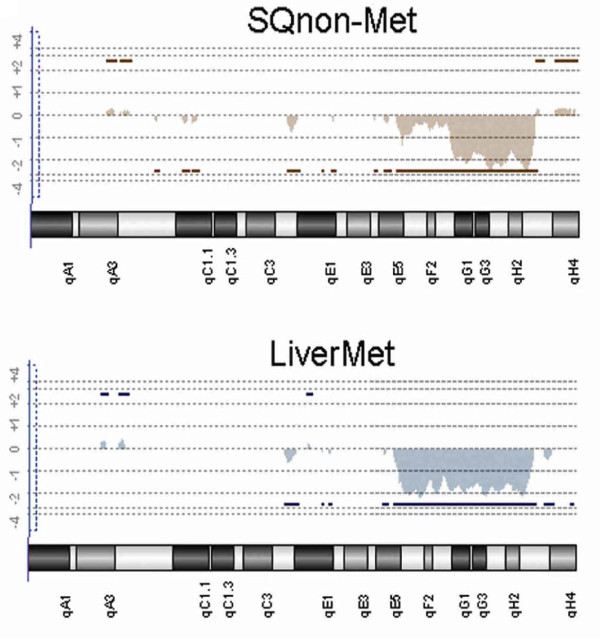
**Chromosome 2 oligonucleotide array CGH plots**. Chromosome 2 plots are shown for one representative non-metastatic (SQnon-Met) and one representative metastatic (LiverMet) tumor. The normalized log2 ratio is on the Y axis, and the chromosome bands are designated on the X axis. Copy number gains and losses are designated by the bars above or below a log2 ratio of 0, respectively. The shaded area reflects the magnitude of the gain or loss. The metastatic tumors show only the larger chromosome 2 deletion (bottom panel), whereas the non-metastatic tumors demonstrate clonal heterogeneity, with the both the smaller deletion, as well as low-level mosaicism for the larger deletion (top panel).

Copy number alterations in tumors were identified by array CGH performed using BAC arrays for all tumors, and oligonucleotide arrays for a subset of tumors. Array CGH using both BAC and oligonucleotide platforms detected multiple gains and losses in both non-metastatic tumors (SQnon-met) and tumors with metastatic potential (SQmet and LiverMet). Both array platforms uncovered similar chromosome imbalances, but the oligonucleotide arrays allowed us to more precisely map regions of gain and loss. Many copy number alterations were common to both groups of tumors, including deletions involving regions of chromosomes 1, 2, 3, 4, 8, 13, 16, 17 18, 19, whole chromosome loss of 14, and gains of chromosome 8 (Figure 1 and Table 1); however, differences in copy number changes between the tumors with and without metastatic potential were also uncovered (Figure 1 and Table 1). One notable difference between the two groups of tumors was a differential region of deletion in distal chromosome 2. Both the metastatic and non-metastatic tumors shared a common 27.4 Mb interstitial deletion of distal chromosome 2, from bands F3 to H3 (139,585,560-167,088,181 bp); however, the metastatic tumors (LiverMet) showed a larger 45 Mb deletion (122,316,740-167,088,181 bp) that extended proximally to band E5 (Figure 2). The non-metastatic tumors showed clonal heterogeneity for both the larger and smaller deletions, with low-level mosaicism for the larger deletion (Figure 2 top), but the clone or clones with the larger deletion were enriched in the metastatic tumors (Figure 2 bottom).

**Table 1:** Summary of acquired copy number changes identified by oligonucleotide array CGH in liver metastases (LiverMet) from NE-10 allografts, and in non-metastasizing (SQnon-met) NE-10 allografts

**Chromosome**	**SQnon-met**	**LiverMet**
1 - loss	*84525268-103082423	84525268-103082423

2 - loss	139585560-167088181	122316740-167088181

3 - loss	11809017-qter	11809017-qter

4 - loss	3010281-131339250	3010281-131339250

4 - gain	N.A.	132282308-149233400

5 - loss	3003879-114937488	N.A.

7 - loss	N.A.	whole chromosome

8 - gain	8397754-17345126	8397754-17345126

8 - gain	20825979-35687899	20825979-35687899

8 - loss	37627333-qter	37627333-qter

10 - gain	79500370-89346058	N.A.

10 - loss	89748171-qter	N.A.

12 - gain	3021012-12590005	N.A.

13 - loss	whole chromosome	3015154-60624834

14 - loss	whole chromosome	whole chromosome

15 - gain	99348507-qter	N.A.

16 - loss	3026317-36861999	whole chromosome

17 - loss	3023355-10795310	whole chromosome

17 - loss	29138819-qter	whole chromosome

18 - loss	35576728-qter	whole chromosome

19 - loss	46971550-qter	46971550-qter

X - gain	145268084-149114686	N.A.

N.A. = not applicable

*Nucleotide sequences are from NCBI build 35

### Differences in gene expression profiles between metastatic and non-metastatic tumors

To obtain differential gene expression profiles between non-metastatic and metastatic NE-10 tumors, the different groups of tumors (SQnon-met, SQmet, and LiverMet) were profiled using gene expression microarrays and analyzed by DIGMAP. A T-test sliding window whole genome scan was performed and plotted by chromosome (see Additional file 2). Based on this analysis, chromosome 2 had the greatest number of regions showing the largest difference in gene expression between metastatic (SQmet and LiverMet) and non-metastatic (SQnon-met) tumors. A detailed T-test sliding window plot of chromosome 2 revealed the highest DFR expression peak in band H4, from 179 to 181 Mb (see Additional file 2). This region is distal to the common region of deletion found in both the metastatic and non-metastatic tumors. Three other smaller peaks were also located slightly more centromeric, from 155 to 168 Mb, within the common region of deletion. Thus, regions of differential gene expression between metastatic and non-metastatic tumors were located both within and distal to the common chromosome 2 deletion; however, we were unable to detect any clusters of genes that demonstrated down-regulation in the metastatic compared to the non-metastatic tumors. There were also no significant differences in gene expression detected in the differential region of deletion on chromosome 2 between the metastatic and non-metastatic tumors.

### Identification of candidate metastasis suppressor genes in chromosome 2 differentially deleted region

Metastasis suppressor genes are expected to show inactivation or decreased activity in metastatic tumors because of genetic or epigenetic changes that result in loss or down-regulation of expression. The finding of a differentially deleted region on chromosome 2, potentially resulting in loss of a metastasis suppressor gene in the metastatic tumors, led us to focus on this region for more detailed analysis. Given that only a subset of genes in the differentially deleted region was represented on the expression arrays, we employed an informatics approach to identify all potential candidate metastasis suppressor genes in the region of interest (chromosome 2 E5-F3; nucleotides 122,316,740-139,585,560). Two parallel bioinformatics filters were designed to carry out this analysis. Out of a total of 146 genes in the region of interest, 11 candidate metastasis suppressor genes were identified based on the function-based filters, while 22 candidates were identified using a filter that employed a cancer expression signature filter using the EXALT database (see Additional file 3). A final list of four candidate genes (*Slc27a2, Mall, Snrpb*, and *Rassf2*) was chosen based on having the highest total score.

### Quantitative RT-PCR of 4 candidate metastasis suppressor genes in chromosome 2 differentially deleted region

**Figure 3:**
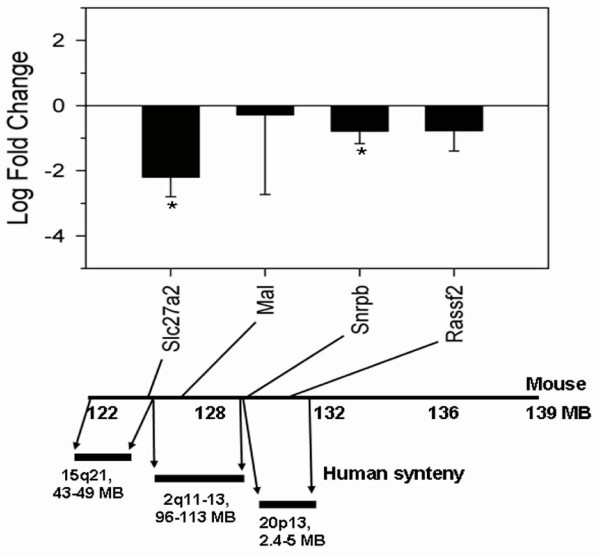
**Relative expression levels between metastatic and non-metastatic NE-10 tumors by RT-PCR**. The X-axis shows four candidate metastasis suppressor genes and the Y-axis shows relative fold change (log2 based) between SQMet and SQnonMet NE-10 tumors (top panel). The relative expression levels are designated by the height of bars, and standard deviation error bars are in the direction of SQMet samples. An asterisk indicates significantly decreased expression in SQMet samples (P < 0.05). The corresponding genomic locations of the four candidate genes on mouse chromosome 2, and the regions of conserved synteny in human, are shown below.

Quantitative RT-PCR for the four candidate metastasis suppressor genes was performed in tumors with metastatic potential (SQMet) and tumors without metastatic potential (SQnon-Met). Metastatic liver tumors were not analyzed due to the presence of contaminating normal liver tissue in some of the tumors. All four candidate metastasis suppressor genes demonstrated decreased expression in the metastasizing tumors compared to the non-metastasizing tumors, with *Slc27a2 *and *Snrpb *showing statistically significant decreased expression in the metastasizing tumors (Figure 3).

## Discussion

Using high-resolution array CGH, we uncovered multiple copy number changes common to both the metastatic and non-metastatic tumors, consistent with a phenotype of genomic instability in this model. Many of the regions of loss overlap with frequent losses that have been observed by conventional CGH and array CGH performed on localized and metastatic human PCa, including human 4q22.3-q31.1, 5q21.1-q21.3, 6q15-q16.2, 8p21.1-p23.1, 10q23.1, 10q25.1-q25.3, 13q14.2-q22.1, 16q12.1-q24.3, 18q12.3-q21.1, 18q21.31-q21.32, 18q22.1-q23 [10-12]. Loss of specific tumor suppressor genes implicated in human PCa, such as *Nkx3-1 *and *Rb1*, was also present in the mouse NE-10 allograft. Other alterations that are frequently seen in human PCa, such as loss of *PTEN*, loss of *CDKN1B*, and gain of *MYC*, were not observed in the NE-10 model.

In addition to copy number alterations common to both the metastatic and non-metastatic tumors, we also observed differences in copy number alterations between these two groups of tumors that could be responsible for their divergent behavior. These differences included loss of chromosome 2 (122.3-139.6 Mb), gain of chromosome 4 (132.2-149.2 Mb), loss of chromosome 7, loss of chromosome 16 (36.9 Mb -qter), loss of chromosome 17 (10.8- 29.1 Mb), and loss of chromosome 18 (3.3-35.6 Mb) in the metastatic, but not the non-metastatic tumors. Our attention was drawn to a differentially deleted region of chromosome 2 for a number of reasons. First, haploinsufficiency or inactivation of metastasis suppressor genes can occur through deletion. We have also detected distal chromosome 2 deletions in approximately 35% of metastatic tumors from multiple independent mice from the original 12T-10 transgenic line (unpublished data), confirming that this is a recurring chromosome abnormality in tumors from this model. In addition, a similar deletion of chromosome 2 has previously been reported in a mouse model of acute promyelocytic leukemia [5]. Also, the larger region of deletion that is enriched in the metastatic tumors demonstrates conserved synteny with the short arm of human chromosome 20 that has been implicated in metastatic PCa [13]. In the mouse model of acute promyelocytic leukemia, loss of one copy of the *Sfpi1 *(*Pu.1*) gene due to the chromosome 2 deletion, and reduced expression of this gene, have been implicated in the progression of leukemia in these mice [14]. The *Sfpi1 *gene is not an obvious candidate metastasis suppressor gene in the NE-10 allograft model, as the gene maps approximately 31 Mb centromeric to the proximal chromosome 2 deletion breakpoint in the metastatic tumors. *Cd82 *(*Kai1*) and Cd44, known metastasis suppressor genes for prostate [15,16], are also located many megabases (~29 Mb and 20 Mb, respectively) proximal to the chromosome 2 deletion. Expression array analyses that we performed also did not uncover candidate metastasis suppressor genes within this differentially deleted region; therefore, we employed a bioinformatics approach to identify potential candidate genes that may be involved in the metastatic behavior of these tumors.

The candidate metastasis suppressor genes in the differential region of deletion of chromosome 2 that we identified, based on prior evidence of metastasis suppressor function and down-regulation in cancer, consisted of *Slc27a2, Mall, Snrpb*, and *Rassf2*. All of these genes demonstrated decreased expression in the metastasizing compared to the non-metastasizing NE-10 tumors by quantitative RT-PCR. *MALL *(*MAL*, T-cell differentiation protein) is a raft-associated integral membrane protein that is involved in membrane trafficking processes. Expression of the MAL protein has been demonstrated in specific types of normal epithelial cells throughout the respiratory system, the gastrointestinal tract, and the genitourinary tract, including strong expression in the ductal and acinar cells of the prostate [17]. Loss of or decreased *MALL *expression, sometimes as the result of DNA methylation of the promoter region, has been found in a variety of benign and malignant epithelial tumors compared to their normal epithelial counterparts, consistent with a role for this protein in tumor suppression [17-21]. Furthermore, MAL has been shown to enhance apoptosis through the Fas pathway, and suppress tumorigenicity, invasion, and motility [21]. Although loss or decreased *MALL *expression has not been evaluated in human prostate cancer, the expression of this protein in normal prostate epithelium and its ability to suppress invasion and motility render it a biologically plausible metastasis suppressor gene for prostate cancer.

*SLC27a2 *encodes a protein that is an isozyme of the long-chain fatty-acid-coenzyme A ligase family, and as such plays a role in lipid biosynthesis and fatty acid degradation. It may also be involved in translocation of long-chain fatty acids across membranes [22]. Long-chain fatty acids participate in many cellular functions and have been implicated as modulators of carcinogenesis, partly through their ability to activate peroxisome proliferator-activated receptors (PPAR). There is evidence that PPAR gamma regulates prostatic epithelial differentiation and may restrict epithelial proliferation; therefore, it is possible that decreased expression of *Slc27a2 *in the NE-10 allograft model could alter the tumor suppressor activity of PPAR gamma and contribute to metastatic behavior.

With regard to *RASSF2 *and *SNRPB*, Goodzari and co-workers found evidence for a prostate cancer metastasis suppressor gene on the short arm of human chromosome 20 [13]. The differential region of chromosome 2 deletion in metastatic compared to non-metastatic tumors in the NE-10 model shows conserved synteny with a region of human 20p. *RASSF2 *and *SNRPB *also map to the short arm of human chromosome 20, although they are telomeric to the most likely metastasis suppressor region of 20p (20p11.23-p12) that was proposed [13]. *SNRPB *encodes a protein that is one of several nuclear proteins that are found in common among U1, U2, U4/U6, and U5 small ribonucleoprotein particles (snRNPs). These snRNPs are involved in pre-mRNA splicing. *RASSF2 *(RAS association domain family 2) is a negative effector of Ras and has been implicated as a tumor suppressor gene in a number of other forms of epithelial cancer, such as colon, gastric, breast, and lung carcinoma [23-25]. The mechanism of inactivation of *RASSF2 *described in these tumors is aberrant promoter methylation, primarily in early tumors. In addition, inactivation of the A isoform of *RASSF2 *by promoter methylation has been found to correlate with a higher frequency of lymph node metastases in patients with nasopharyngeal carcinoma [26].

In summary, using an approach based on bioinformatic filters applied to array CGH data on a divergent metastasizing and non-metastasizing mouse model of PCa, we have identified genes that are biologically plausible metastasis suppressor genes. Our studies have identified an association between deletion with decreased expression of these genes and the metastasizing NE-10 model, suggesting a biological role for these genes in the metastatic process. We believe the divergence in behavior of the allograft can be attributed to clonal heterogeneity, with both metastasizing and non-metastasizing clones present in early allograft passages, but with selection for a non-metastasizing clone or clones after multiple in *vivo *passages. This selection could be due to preferential subcutaneous growth of the non-metastasizing compared to the metastasizing component of the allograft, such that over time, a larger and larger percentage of the allograft consists of the non-metastasizing population. It is possible that the clone or clones with the larger chromosome 2 deletion are enriched in the metastatic tumors because the differentially deleted region itself is responsible for the metastatic behavior, either alone or in combination with other genetic alterations, or that the larger deletion is simply a marker for a clone that is selected for because of the presence of some other alteration that confers metastatic potential. Additional studies are needed to prove that a gene or genes within the differentially deleted region of chromosome 2 are responsible for suppression of metastatic behavior.

## Conclusion

We have taken advantage of a mouse allograft model of PCa (NE-10) with divergent metastasizing and non-metastasizing behavior to identify regions of the genome that potentially harbor metastasis suppressor genes. Using a combination of genomics and bioinformatics approaches, we identified candidate genes from a differentially deleted region on mouse chromosome 2 between the metastasizing and non-metastasizing allograft lines. The genes presented here are candidates for further studies to determine their functional role in inhibiting metastases in the NE-10 allograft model and human PCa.

## List of abbreviations

NE-10: mouse allograft established from a primary tumor from the LPB-Tag mouse model of prostate cancer; PCa: prostate cancer; SQnon-met: non-metastasizing subcutaneous allograft; SQmet: metastasizing subcutaneous allograft; LiverMet: liver metastases; CGH: comparative genomic hybridization; BAC: bacterial artificial chromosome; DFRs: differential flagged regions; TTSW: T-test sliding window.

## Competing interests

The authors declare that they have no competing interests.

## Authors' contributions

YY interpreted the expression array results, conducted the bioinformatics, directed and interpreted the RT-PCR experiments, and helped draft the manuscript. SN participated in the expression array experiments and helped revise the manuscript. TC maintained the allografts and harvested tissues. CN provided oversight of the expression array experiments. TR performed the quantitative RT-PCR experiments. RJM provided the allograft model, designed the expression array experiments, and helped draft the manuscript. KDT conducted and interpreted the array CGH experiments, and drafted the manuscript. All authors read and approved the final manuscript.

## Supplementary Material

Additional file 1 **Weighted scoring for the selection of candidate genes in 2E5-2F3**. Detailed description of the weighted scoring method that was used to select candidate suppressor genes in the differentially deleted region of chromosome 2 between metastasizing and non-metastasizing allografts. Click here for file

Additional file 2 **T-test sliding window whole genome and chromosome 2 analysis**. Comparison of expression array results between metastatic and non-metastatic allografts using a T-test sliding window analysis. Click here for file

Additional file 3 **Complete candidate gene list**. Complete list of candidate genes from the 2E5-2F3 identified from the function-based bioinformatics filter and the cancer expression signature filter. Click here for file
