# Supplementary material for: Candidate metastasis suppressor genes uncovered by array comparative genomic hybridization in a mouse allograft model of prostate cancer
Source: Mol Cytogenet. 2009 Sep 26;2:18. doi: 10.1186/1755-8166-2-18 (PMC2761934; doi:10.1186/1755-8166-2-18)
Supplement: Additional file 1 — Weighted scoring for the selection of candidate genes in 2E5-2F3. Detailed description of the weighted scoring method that was used to select candidate suppressor genes in the differentially deleted region of chromosome 2 between metastasizing and non-metastasizing allografts. [file 1755-8166-2-18-S1.DOC]

**Weighted scoring for the selection of candidate genes in 2E5-2F3**

The 146 known genes in the chromosome 2 DFR were analyzed by two filters: the function-based filter and the cancer expression signature-based filter. A weighted score was assigned to each gene at every step of each filter.

The function-based filter utilized the gene ontology database (GO db), Ingenuity pathway analysis (IPA, http://www.ingenuity.com/), and PubMed searches. If a gene had a hit in any one of these databases for a metastasis suppressor term designated by us, a score of one was added to the gene. We chose the following terms because they are conserved across known metastasis suppressor genes: growth, proliferation, adhesion, cytoskeletal, and actin. A total score was calculated for each gene after searching all three databases. The sign of the score was determined by information obtained from PubMed. If evidence of down-regulation of a candidate gene was reported in high-grade tumors or metastatic tumors, a negative sign was prefixed to the gene score. Eleven candidate genes were selected based on final total weight score cutoff of <-3.

The cancer expression signature filter utilized the EXALT database that contains thousands of pre-computed expression signatures from published cancer studies (ref). The 146 gene symbols were used to query this signature database, and only signatures containing input genes with down-regulation in tumor samples were retrieved. Scores were assigned to each gene based on the number of signature hits. A total of 22 candidate genes were selected with scores greater than 0.

The gene lists from the functional filter and the EXALT filter were combined and the absolute values of total weight scores were added together. The final top 4 candidate genes (*Slc27a2, Mal, Snrpb*, and *Rassf2*) were selected because they had the highest scores (>7). Two of the genes (*Rassf2* and *Mal*) were identified by both of the parallel filters, while the other two (*Slc27a2* and *Snrpb*) were identified by the cancer expression signature filter only.
