# Supplementary material for: Candidate metastasis suppressor genes uncovered by array comparative genomic hybridization in a mouse allograft model of prostate cancer
Source: Mol Cytogenet. 2009 Sep 26;2:18. doi: 10.1186/1755-8166-2-18 (PMC2761934; doi:10.1186/1755-8166-2-18)
Supplement: Additional file 2 — T-test sliding window whole genome and chromosome 2 analysis. Comparison of expression array results between metastatic and non-metastatic allografts using a T-test sliding window analysis. [file 1755-8166-2-18-S2.PPT]

## Slide 1
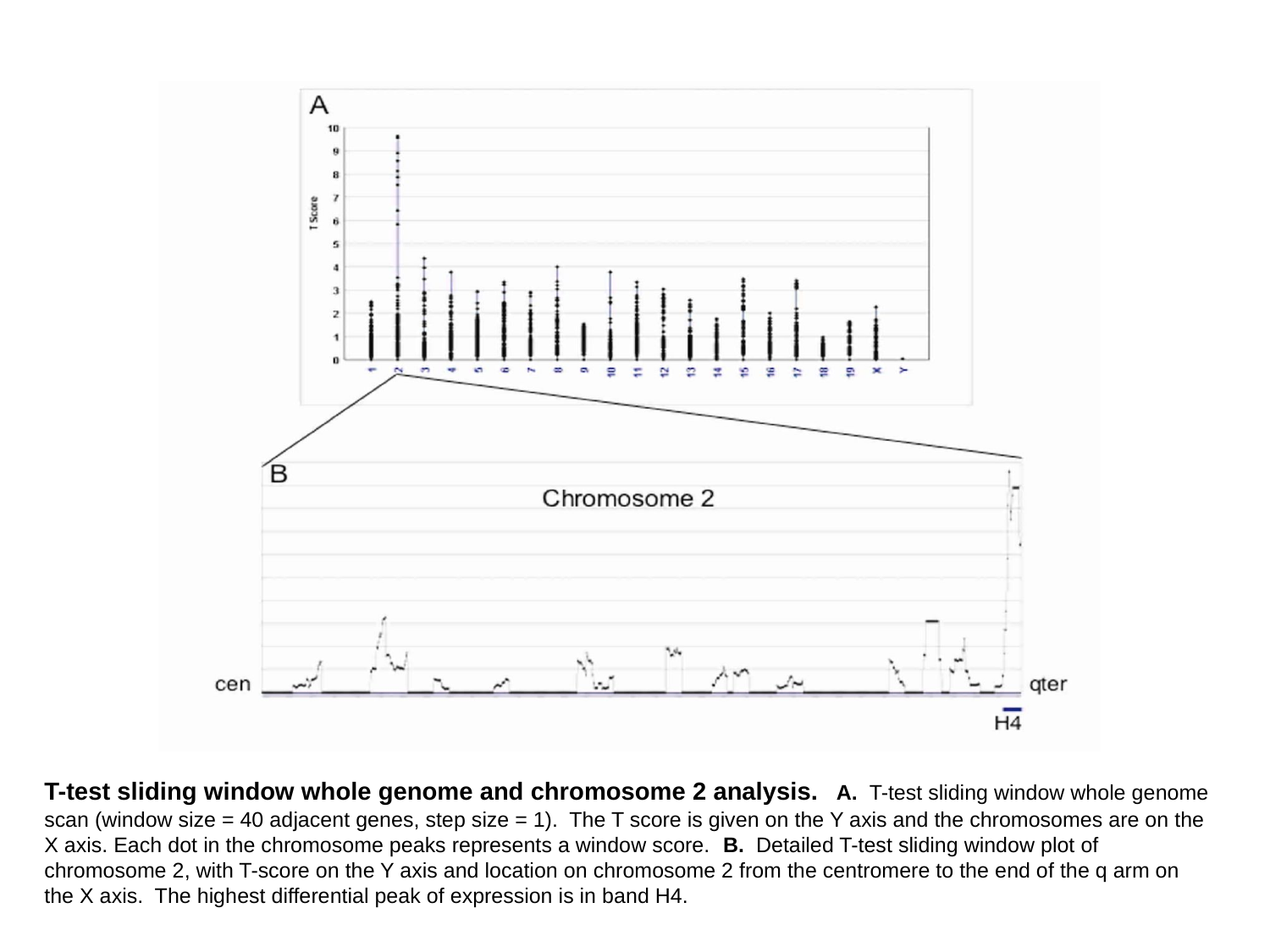

T-test sliding window whole genome and chromosome 2 analysis. A. T-test sliding window whole genome scan (window size = 40 adjacent genes, step size = 1). The T score is given on the Y axis and the chromosomes are on the X axis. Each dot in the chromosome peaks represents a window score. B. Detailed T-test sliding window plot of chromosome 2, with T-score on the Y axis and location on chromosome 2 from the centromere to the end of the q arm on the X axis. The highest differential peak of expression is in band H4.
