# Supplementary material for: Candidate metastasis suppressor genes uncovered by array comparative genomic hybridization in a mouse allograft model of prostate cancer
Source: Mol Cytogenet. 2009 Sep 26;2:18. doi: 10.1186/1755-8166-2-18 (PMC2761934; doi:10.1186/1755-8166-2-18)
Supplement: Additional file 3 — Complete candidate gene list. Complete list of candidate genes from the 2E5-2F3 identified from the function-based bioinformatics filter and the cancer expression signature filter. [file 1755-8166-2-18-S3.DOC]

**Additional Table 1.** Complete candidate gene list

| Gene | Signature- based score^a^ | Function- based score^b^ | Total score^c^ |
| --- | --- | --- | --- |
| *Rassf2* | 10 | 4 | 14 |
| *Mall* | 5 | 4 | 9 |
| *Snrpb* | 8 |  | 8 |
| *Slc27a2* | 8 |  | 8 |
| *Fbn1* | 5 | 2 | 7 |
| *Bmp2* | 7 |  | 7 |
| *Slc30a4* |  | 7 | 7 |
| *Ttl* |  | 7 | 7 |
| *Gabpb1* | 6 |  | 6 |
| *Avp* | 6 |  | 6 |
| *Pdyn* | 6 |  | 6 |
| *Chgb* | 6 |  | 6 |
| *Sn* |  | 6 | 6 |
| *2310032D16Rik* | 5 |  | 5 |
| *Cpxm1* | 5 |  | 5 |
| *1700037H04Rik* | 5 |  | 5 |
| *Hdc* | 5 |  | 5 |
| *Sqrdl* | 5 |  | 5 |
| *Cri1* |  | 5 | 5 |
| *Bcl2l11* |  | 5 | 5 |
| *Rpo1-2* | 4 |  | 4 |
| *Fgf7* | 4 |  | 4 |
| *Dut* |  | 4 | 4 |
| *Dusp2* |  | 4 | 4 |
| *Zc3h8* |  | 4 | 4 |
| *Nol5a* | 3 |  | 3 |
| *Prnp* | 3 |  | 3 |
| *Cdc25b* | 2 |  | 2 |
| *Ptpra* | 2 |  | 2 |
| *Bub1* | 1 |  | 1 |
|  |  |  |  |

^a^Signature-based scores were assigned to each gene based on the number of signature hits in EXALT signature database with down-regulation in tumor samples.

^b^Function-based scores were absolute values calculated for each gene after searching all three annotation databases (Gene Ontology database, Ingenuity Pathway Analysis and PubMed).

^c^Total Scores are total weight scores from both signature-based scores and function-based scores.
